# Bacteria from the Midgut of Common Cockchafer (*Melolontha melolontha* L.) Larvae Exhibiting Antagonistic Activity Against Bacterial Symbionts of Entomopathogenic Nematodes: Isolation and Molecular Identification

**DOI:** 10.3390/ijms21020580

**Published:** 2020-01-16

**Authors:** Marcin Skowronek, Ewa Sajnaga, Małgorzata Pleszczyńska, Waldemar Kazimierczak, Magdalena Lis, Adrian Wiater

**Affiliations:** 1 Laboratory of Biocontrol, Application and Production of EPN, Centre for Interdisciplinary Research, Faculty of Biotechnology and Environmental Sciences, John Paul II Catholic University of Lublin, ul. Konstantynów 1J, 20-708 Lublin, Poland; esajnaga@kul.pl (E.S.); wklublin@tlen.pl (W.K.); mdybala@kul.pl (M.L.); 2 Department of Industrial and Environmental Microbiology, Faculty of Biology and Biotechnology, Maria Curie-Skłodowska University, ul. Akademicka 19, 20-033 Lublin, Poland; mpleszcz@poczta.umcs.lublin.pl

**Keywords:** *Melolontha melolontha*, midgut microbiota, entomopathogenic nematodes, bacterial interactions, *Xenorhabdus*, *Photorhabdus*

## Abstract

The mechanisms of action of the complex including entomopathogenic nematodes of the genera *Steinernema* and *Heterorhabditis* and their mutualistic partners, i.e., bacteria *Xenorhabdus* and *Photorhabdus*, have been well explained, and the nematodes have been commercialized as biological control agents against many soil insect pests. However, little is known regarding the nature of the relationships between these bacteria and the gut microbiota of infected insects. In the present study, 900 bacterial isolates that were obtained from the midgut samples of *Melolontha melolontha* larvae were screened for their antagonistic activity against the selected species of the genera *Xenorhabdus* and *Photorhabdus*. Twelve strains exhibited significant antibacterial activity in the applied tests. They were identified based on 16S rRNA and *rpoB*, *rpoD*, or *recA* gene sequences as *Pseudomonas chlororaphis*, *Citrobacter murliniae*, *Acinetobacter calcoaceticus*, *Chryseobacterium lathyri*, *Chryseobacterium* sp., *Serratia liquefaciens*, and *Serratia* sp. The culture filtrate of the isolate *P. chlororaphis* MMC3 L3 04 exerted the strongest inhibitory effect on the tested bacteria. The results of the preliminary study that are presented here, which focused on interactions between the insect gut microbiota and mutualistic bacteria of entomopathogenic nematodes, show that bacteria inhabiting the gut of insects might play a key role in insect resistance to entomopathogenic nematode pressure.

## 1. Introduction

Coleoptera is the largest order of insects, representing over 400,000 of known species. During evolution, representatives of this order have colonized diverse ecological niches and climate zones. The most abundant (over 30,000 species) family of these insects is Scarabaeidae, with characteristic lamellate club antennae in imagines [1]. The evolutionary success of scarabs lies in the activity of the gut microbiota of their larvae, which allows for them to feed on a wide range of low energy foods, grass roots, and organic matter, with inconsiderable competition from other insects. Hence, scarab larvae exploit a variety of niches, which range from rotting organic matter and dead tree trunks to freshly growing roots. Using these resources, they have easily become pests in agriculture and forestry. Of the approximately 150 scarab species that were recorded in Central Europe, damage is predominantly caused by only four native species: *Melolontha melolontha*, *Melolontha hippocastani*, *Amphimallon solstitialis*, and *Phyllopertha horticola* [2]. These pests are difficult to control due to the cryptic position of larvae in the soil and the usually nocturnal activity of adults [3].

Entomopathogenic nematodes (EPN) of the genera *Steinernema* and *Heterorhabditis* (Nematoda: Rhabditida) are effective biocontrol agents against soil-dwelling stages of many insect pests [4,5,6]. They are safe for vertebrates, plants, and numerous invertebrates [7,8]. In recent years, significant progress in applying these bioagents to reduce the populations of Scarabaeidae pests was observed [9,10,11,12]. Entomopathogenic nematodes are symbiotically associated with entomopathogenic bacteria (EPB) *Xenorhabdus* spp. and *Photorhabdus* spp. EPB are motile, gram-negative, non-spore-forming, facultatively anaerobic rods of the family *Enterobacteriaceae*. They live in the intestinal lumen of infective juveniles (the only free-living stage of EPN) and in the body cavity of the infected insects [13]. Infective juveniles migrate to the hemocoel and release bacterial symbionts, which multiply quickly causing a lethal bacteremia within 24–48 h, after entering the body of an insect through the orifices of the respiratory and digestive systems [14,15]. However, findings regarding *Steinernema carpocapsae* indicate that some of the bacteria (*Xenorhabdus nematophila*) transported by nematodes may be released in the insect gut as early as several hours after the entrance of the parasites into the gastrointestinal canal [12]. It has also been found that, during early infection, *Photorhabdus* bacteria specifically proliferate in the midgut, where they release toxins and a metalloprotease that destroy the midgut epithelium [16]. A growing bacterial population provides an optimal environment for the rapid development of the nematodes. Compounds that are secreted by these bacteria, such as lytic enzymes and substances with antimicrobial properties, give EPB a competitive advantage, which facilitates their rapid invasion of an attractive environment, despite the initial presence of the species-rich indigenous microbiota of the insect gut [17,18].

Entomopathogenic nematodes with their mutualistic bacteria and infected insects have become the subject of numerous research studies, which have already provided considerable data, e.g., on the mechanisms of pathogenesis, insect immunity, and the ecological aspects of the biological complex [19]. It can be expected that the interactions between the mutualistic bacteria of EPN and the gut-associated bacteria of the infected insects represent a highly important component of the complicated nematode-bacterium-insect complex, but they have not been investigated in detail. Previous studies in this area primarily focused on the antibacterial activities of EPB. To date, there have been no studies on antagonistic mechanisms that would work in the opposite direction, i.e., growth inhibition of the nematode bacterial symbionts by bacteria of the insect gut microbiota. This type of interaction is to be expected, based on the fact that the body of the insect harbors two groups of bacteria with conflicting interests when a nematode larva has released EPB. The optimal habitat for the insect gut microbiota is the digestive system of a living host, while developing EPB lead to a rapid death of the insect. It is possible that some of the gut bacteria are capable of producing substances that have an antagonistic action against EPB, thus protecting both the intestinal microbiota and the entire body of the insect. This type of bacterial activity might substantially reduce the effectiveness of biopesticides containing entomopathogenic nematodes. The isolation and identification of insect gut bacteria, which exhibit antimicrobial activity against *Xenorhabdus* spp. or *Photorhabdus* spp., would allow for a much better understanding of the mechanism of EPN infection of insects and the bacterial interactions that occur during this process.

The objective of this study was to isolate and identify the bacteria colonizing the midgut of the common cockchafer *M. melolontha*, which exhibit antibacterial activity against selected species of the genera *Xenorhabdus* and *Photorhabdus*.

## 2. Results

### 2.1. Isolation of Bacteria from the Midgut of M. melolontha Larvae

Sixty samples of the midgut of the second and third instar *M. melolontha* larvae (L2 and L3) were used in the study. The guts were sampled from six groups of larvae. The first group comprised the specimens that had been freshly collected in the natural habitat. The other five groups of larvae were subjected to initial 12-day exposure to entomopathogenic nematodes and ten live larvae from each group were selected for further analyses. This procedure aimed at increasing the probability of acquisition of bacterial isolates with the ability to inhibit the growth of bacteria colonizing the entomopathogenic nematodes. The nematode pressure can be regarded as strong, since the incubation of *M. melolontha* in the presence of EPN resulted in the death of 42.5% of larvae in the presence of *Heterorhabditis megidis*, as well as 55%, 52.5%, 25%, and 35% of larvae that were exposed to *Steinernema arenarium*, *Steinernema bicornutum*, *Steinernema carpocapsae*, and *Steinernema silvaticum*, respectively.

In total, 900 bacterial strains were isolated from the gut samples (15 strains from each gut). The isolation and growth inhibition assays were carried out in aerobic conditions in the case of half of the obtained strains (i.e., 450) and in microaerobic conditions in the case of the other half since the aerobic conditions in the midgut of scarab larvae may vary [20].

### 2.2. Screening of Midgut Bacteria with Antagonistic Activity Against EPB

**Figure 1:**
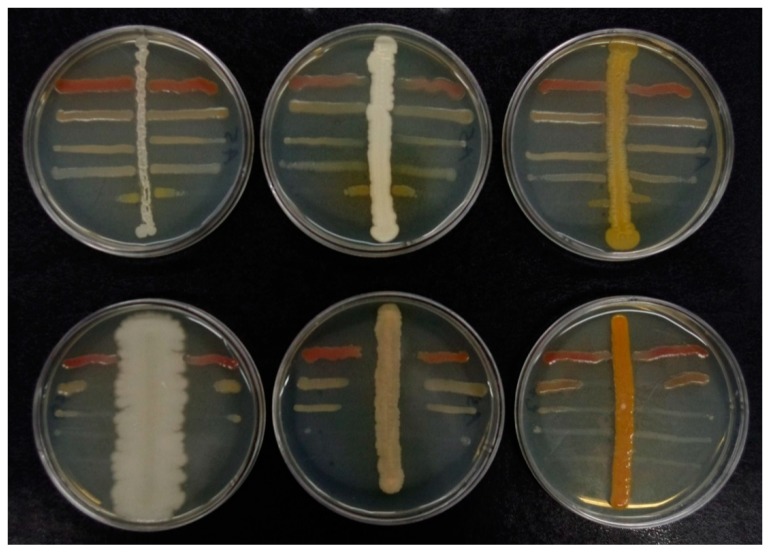
Interactions between *M. melolontha* midgut isolates and nematode bacteria in cross-streak test. Horizontal streaks—five entomopathogenic bacteria (EPB) strains, vertical streak—midgut isolate. Top row—no inhibition effect; bottom row—isolates with high antibacterial activity against EPB.

In the first stage of the investigations, the antibacterial activities of the isolates were analyzed while using cross-streak tests (Figure 1). Thirty-eight isolates inhibited the growth of the selected EPB species, i.e., *Photorhabdus temperata*, *Xenorhabdus kozodoii*, *Xenorhabdus bovienii*, *Xenorhabdus nematophila*, and *Xenorhabdus budapestensis* (Table 1). Twenty-three and fifteen strains with this ability were isolated in the aerobic and microaerobic conditions, respectively. The greatest number of positive isolates (12) was obtained from the midgut of larvae that were exposed to *H. megidis*. Some isolates completely inhibited the growth of the symbiotic bacteria of nematodes over the entire surface of the Petri dishes in the cross-streak tests. In most cases, the lowest susceptibility to growth inhibition characterized *P. temperata* (Table 1).

**Figure 2:**
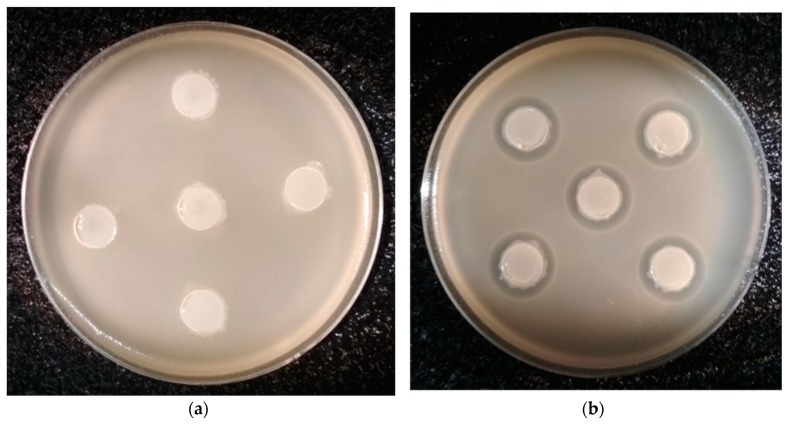
Interactions between *M. melolontha* midgut isolates and nematode bacteria in a modified agar well diffusion test. (**a**) Isolate incapable of inhibition of the growth of EPB. (**b**) MMC3 L3 04 isolate showing antagonistic activity against *X. kozodoii*.

Next, the selected isolates were subjected to modified agar well diffusion tests to confirm their antibacterial activity. Twelve isolates were shown to have the ability to inhibit the growth of the symbiotic bacteria of nematodes. The highest antibacterial activity was detected for the isolate MMC3 L3 04 (Figure 2 and Table 2).

### 2.3. Identification of Selected Midgut Isolates from M. melolontha Larvae

Subsequently, isolates that inhibit the growth of the *Xenorhabdus* and *Photorhabdus* bacteria in both the cross-streak tests and the modified agar well diffusion tests were identified with the use of molecular methods. Preliminary identification was based on 16S rRNA gene sequence analysis. Nearly full-length 16S rDNA sequences of all tested isolates were determined while using a pair of universal primers 27F and 1492R. Additionally, the *rpoD*, *rpoB* or *recA* gene sequences were analyzed while using genus specific primers (Table 3).

Based on the 16S rDNA and protein-coding housekeeping gene sequences, six isolates were identified as *Pseudomonas chlororaphis* and single isolates represented *Citrobacter murliniae*, *Acinetobacter calcoaceticus*, *Chryseobacterium lathyri*, *Serratia liquefaciens*, *Serratia* sp. and *Chryseobacterium* sp. (Table 4). The molecular identification of the isolates was supported by phylogenetic analysis, whose results are shown in supplementary data (Supplementary Materials Figures S1–S6).

### 2.4. Detailed Evaluation of Antibacterial Activity of Selected Gut Isolates from M. melolontha Larvae

**Figure 3:**
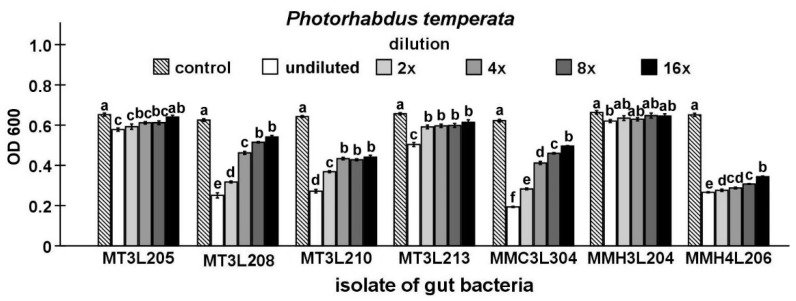
Antagonistic activities of different dilutions of culture supernatants from selected *M. melolontha* midgut isolates against *P. temperata* shown by Maximum Inhibitory Dilution (MID) tests. The same letters mean no significant differences between the activities of supernatants from the same midgut isolate.

**Figure 4:**
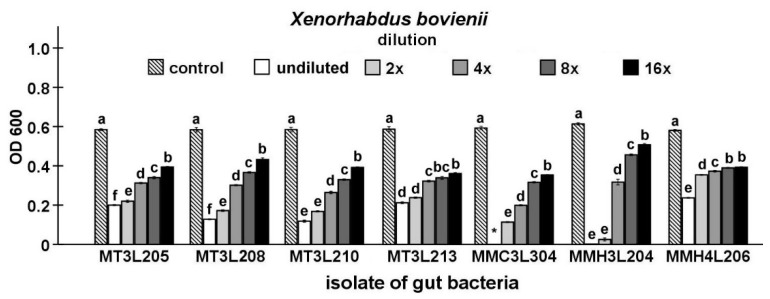
Antagonistic activities of different dilutions of culture supernatants from selected *M. melolontha* midgut isolates against *X. bovienii* shown by MID tests. The same letters mean no significant differences between the activities of supernatants from the same midgut isolate. *—no bacterial growth.

**Figure 5:**
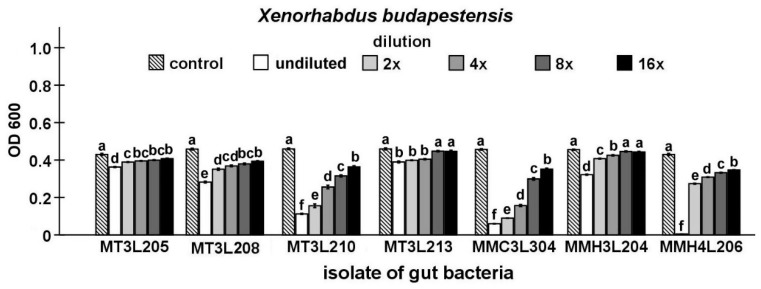
Antagonistic activities of different dilutions of culture supernatants from selected *M. melolontha* midgut isolates against *X. budapestensis* shown by MID tests. The same letters mean no significant differences between the activities of supernatants from the same midgut isolate.

**Figure 6:**
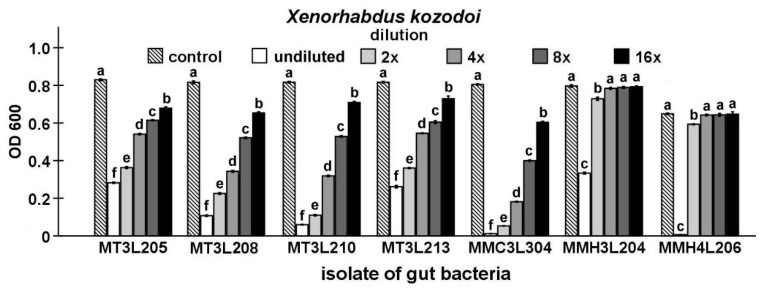
Antagonistic activities of different dilutions of culture supernatants from selected *M. melolontha* midgut isolates against *X. kozodoi* shown by MID tests. The same letters mean no significant differences between the activities of supernatants from the same midgut isolate.

**Figure 7:**
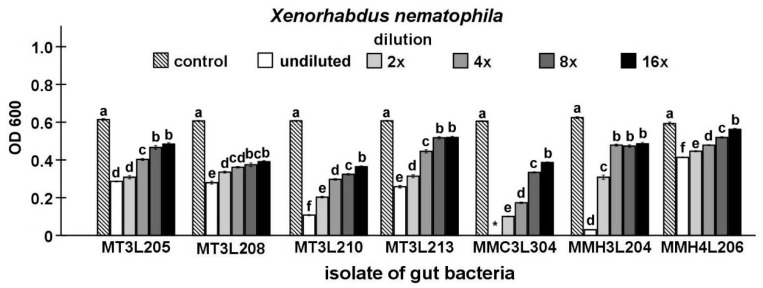
Antagonistic activities of different dilutions of culture supernatants from selected *M. melolontha* midgut isolates against *X. nematophila* shown by MID tests. The same letters mean no significant differences between the activities of supernatants from the same midgut isolate. *—no bacterial growth.

Finally, the antibacterial activity of the isolates that were identified in the study was compared while using Maximum Inhibitory Dilution (MID) tests. In this stage of the study, seven strains, including five strains that represent *P. chlororaphis* and two from the genus *Chryseobacterium*, were found to be able to inhibit the growth of the symbiotic bacteria of nematodes (Figure 3, Figure 4, Figure 5, Figure 6 and Figure 7).

The undiluted supernatant of the *P. chlororaphis* MMC3L304 isolate caused the strongest inhibition of *P. temperata* growth (*t*-test t_18_ = 58.86, *p* < 0.001). In the undiluted supernatant groups, the supernatant of the *C. lathyri* MMH3L204 isolate exerted the lowest inhibitory effect on *P. temperata* growth (*t*-test t_18_ = 3.68, *p* < 0.01). The supernatant dilution had a statistically significant influence on *P. temperata* growth (ANOVA, F_5,54_ ≥ 9.40, *p* < 0.001). The supernatant of the *Chryseobacterium* sp. MMH4L206 isolate (*t*-test t18 = 34.72, *p* < 0.001) exerted the highest inhibitory effect on *P. temperata* growth in the 16-fold diluted supernatant group. This dilution of the *P. chlororaphis* MT3L205 and *C. lathyri* MMH3L204 supernatants had no significant influence on *P. temperata* growth (Figure 3).

The undiluted supernatant of the *P. chlororaphis* MMC3L304 isolate caused the complete inhibition of *X. bovienii* growth (*t*-test, t_18_ = 111.31, *p* < 0.001). In the undiluted supernatant groups, the supernatant of the *P. chlororaphis* MT3L213 isolate exerted the lowest inhibitory effect on *X. bovienii* growth (*t*-test t_18_ = 27.29, *p* < 0.001). The supernatant dilution had a statistically significant influence on *X. bovienii* growth (ANOVA, F_5,54_ ≥ 343.52, *p* < 0.001). The supernatant of the *P. chlororaphis* MT3L205 isolate (*t*-test t_18_ = 36.66, *p* < 0.001) exerted the highest inhibitory effect on *X. bovienii* growth, while the lowest inhibitory effect was exhibited by the *C. lathyri* MMH3L204 isolate supernatant (*t*-test t_18_ = 10.55, *p* < 0.001) in the 16-fold diluted supernatant group (Figure 4).

The undiluted supernatant of the *P. chlororaphis* MMC3L304 isolate caused the strongest inhibition of *X. budapestensis* growth (*t*-test, t_18_ = 100.31, *p* < 0.001). In the undiluted supernatant groups, the supernatant of the *P. chlororaphis* MT3L213 isolate exerted the lowest inhibitory effect on *X. budapestensis* growth (*t*-test t_18_ = 9.42, *p* < 0.001). The supernatant dilution had a statistically significant influence on *X. budapestensis* growth (ANOVA, F_5,54_ ≥ 43.29, *p* < 0.001). The supernatant of the *P. chlororaphis* MMC3L304 isolate (*t*-test t_18_ = 16.14, *p* < 0.001) exerted the highest inhibitory effect on *X. budapestensis* growth in the 16-fold diluted supernatant group. There was no significant effect of the 8- and 16-fold *P. chlororaphis* MT3L213 and *C. lathyri* MMH3L204 supernatant dilutions on *X. budapestensis* growth (Figure 5).

The undiluted supernatant of the *P. chlororaphis* MMC3L304 isolate caused the strongest inhibition of *X. kozodoi* growth (*t*-test t_18_ = 222.50, *p* < 0.001). In the undiluted supernatant groups, the supernatant of the *C. lathyri* MMH3L204 isolate exerted the lowest inhibitory effect on *X. kozodoi* growth (*t*-test t_18_ = 54.79, *p* < 0.001). The supernatant dilution had a statistically significant influence on *X. kozodoi* growth (ANOVA, F_5,54_ ≥ 968.90, *p* < 0.001). The supernatant of the *P. chlororaphis* MMC3L304 isolate (t-test t_18_ = 31.49, *p* < 0.001) exerted the highest inhibitory effect on *X. kozodoi* growth in the 16-fold diluted supernatant group. There was no significant effect of the 4-, 8-, and 16-fold *C. lathyri* MMH3L204 and *Chryseobacterium* sp. MMH4L206 supernatant dilutions on *X. kozodoi* growth (Figure 6).

The undiluted supernatant of the *P. chlororaphis* MMC3L304 isolate caused the complete inhibition of *X. nematophila* growth (*t*-test, t_18_ = 260.45, *p* < 0.001). In the undiluted supernatant groups, the supernatant of the *Chryseobacterium* sp. MMH4L206 isolate exerted the lowest inhibitory effect on *X. nematophila* growth (*t*-test t_18_ = 19.58, *p* < 0.001). The supernatant dilution had a statistically significant influence on *X. nematophila* growth (ANOVA, F_5,54_ ≥ 204.68, *p* < 0.001). The supernatant of the *P. chlororaphis* MT3L210 isolate (*t*-test t_18_ = 55.19, *p* < 0.001) exerted the highest inhibitory effect on *X. nematophila* growth, while the lowest inhibitory effect was exhibited by the *Chryseobacterium* sp. MMH4L206 isolate supernatant (*t*-test t_18_ = 2.98, *p* < 0.05) in the 16-fold diluted supernatant group (Figure 7).

As shown above, one of the isolates, i.e., *P. chlororaphis* MMC3 L3 04, exhibited the strongest antagonistic properties against the tested EPB. This strain was isolated under microaerobic conditions from the midgut of a larva that was exposed to *S. carpocapsae* infective juveniles. The culture filtrate of this bacterium significantly inhibited the growth of all *Xenorhabdus* species, with the strongest effect on *X. bovienii* and *X. nematophila*, which was evidenced by the complete inhibition of bacterial growth by the undiluted filtrate (Figure 4 and Figure 7).

## 3. Discussion

In the past years, considerable new information regarding the interactions between mutualistic bacteria of entomopathogenic nematodes and gut-associated bacteria of insects has been provided; however, the findings had a one-sided and limited character. To date, there has only been a unidirectional relationship imposed by EPB through the production of a number of substances that inhibit the growth of insect gut bacteria. It is known that the bacteriocins produced by *Xenorhabdus* and *Photorhabdus* bacteria exhibit strong growth-inhibitory activity against other EPB or bacteria from the host. For example, xenorhabdicin that is produced by *X. nematophila* inhibits the growth of other species of *Xenorhabdus* as well as *P. luminescens* and bacteria of the genus *Proteus* [27]. Similarly, *Photorhabdus* sp. produces photorhabdicin and lumicin-bacteriocins, which are active against other *Photorhabdus* strains and *Escherichia coli* [28,29]. In addition to bacteriocins, EPB generate an array of antimicrobial secondary metabolites, many of which have a wider scope of activity than bacteriocins [30,31]. As bacteria proliferate in the insect’s body, their antibiotic activity steadily grows to reach a maximum between days 3 and 5 of infection, i.e., when the host is already dead [32,33].

The method of selection that was applied in the study helped to isolate strains with antagonistic effects against *Xenorhabdus* and *Photorhabdus* bacteria in the material containing an extremely species-rich insect midgut microbiota. The cross-streak tests used in the initial stage are relatively quick to perform and allow for preliminary analysis of a large number of isolates. The next two stages facilitated the analysis of the antibacterial properties of the isolates with greater precision. Evidently, only some of the strains that were selected by the cross-streak tests had their activity confirmed by the other two tests. This might have been related to the different types of bacterial growth in each test. In the cross-streak tests, the bacteria grew on the surface, whereas, in our modification of the agar well diffusion tests, they were suspended in agar medium. We used a modified version of the agar well diffusion method, as we found that it yielded substantially larger growth inhibition zones in the case of the tested strains, which facilitated the identification of isolates with antibacterial properties. The modification consisted in introduction of LB agar medium inoculated with the tested bacterial isolates into the wells instead of the culture filtrate of these isolates (Materials and Methods 4.6.2).

In the last test, i.e., MID, contrary to the previous ones, there was no direct interaction between the investigated bacterial groups, as bacterium-free culture filtrates were used. This might have contributed to the detection of the antagonistic activity against *Xenorhabdus* and *Photorhabdus* in only seven isolates at this stage. Another explanation of such results might also be the loss of antibacterial activity during storage: the cross-streak tests were carried out immediately after the isolation of the bacteria from larval midguts, whereas the subsequent tests were performed while using freeze-stored bacteria.

As shown in Table 1, most of the isolates displaying antagonistic properties against EPB (23 of the 38 isolates that were obtained in the first stage of the study) were isolated in aerobic conditions. However, the *P. chlororaphis* MMC3 L3 04 strain, which exhibited the highest antibacterial activity in most assays, was isolated in microaerobic conditions. It is noteworthy that the vast majority of the selected strains with antagonistic properties, including all strains identified taxonomically, exhibited the ability to grow in aerobic conditions (data not shown), although some of them were isolated in microaerobic conditions. Therefore, it can be assumed that higher efficiency of screening of the bacteria from the midgut of *M. melolontha* larvae in terms of their antibacterial activity against *Xenorhabdus* and *Photorhabdus* species can be achieved in aerobic conditions.

Comparative analyses of 16S rRNA gene sequences are useful for the classification of cultured microorganisms, especially given the established taxonomic thresholds [34]. However, it is known that the sequencing of the 16S rRNA gene is only not sufficient for the identification of most bacteria at the species level [35,36]. We analyzed the sequences of 16S rRNA genes and those coding for proteins with conserved functions i.e., *rpoB*, *rpoD*, and/or *recA*, which efficiently supplemented the 16S rRNA gene-based identification of the bacteria to reliably identify the isolates at the species level [22,23,24,25,26]. As shown in the present study, the 12 analyzed bacterial isolates represented five genera, namely, *Pseudomonas*, *Citrobacter*, *Serratia*, *Acinetobacter*, and *Chryseobacterium* (Table 4, Figures S1–S6). Half of the isolates with strong antagonistic activity against EPB were identified as *P. chlororaphis*. Four of them were isolated from the midgut of the same *M. melolontha* larva, while the other two were isolated from other specimens collected from different locations at different times (Table 1). The *P. chlororaphis* strains were isolated from the larvae in different developmental phases, i.e., four isolates were obtained from a stage L2 larva and the other two were isolated from two L3 larvae. In most cases, the *P. chlororaphis* strains were characterized by a stronger ability to inhibit the growth of the symbiotic bacteria of nematodes than the other isolates. These results suggest that *P. chlororaphis* bacteria may be an important factor inhibiting the growth of bacteria from the genera *Xenorhabdus* and *Photorhabdus* also in in vivo conditions, i.e., in the organism of *M. melolontha*.

The presence of the *Pseudomonas* sp. bacteria has been repeatedly detected in samples that were collected from Scarabaeidae larvae, e.g., in the midgut of *M. hippocastani* L3 larvae [37], in the hindgut of *Holotrichia parallela* L3 larvae [38], or in the midgut of 3rd instar *Protaetia brevitarsis* larvae, where these bacteria were the most dominant genera [39]. *Pseudomonas* sp. bacteria have also been isolated in studies on their insecticidal activity [40]. To date, the ability of *P. chlororaphis* to inhibit the growth of *Photorhabdus* or *Xenorhabdus* bacteria has not been shown, but its antibacterial activity against other species, e.g., *Clavibacter michiganensis* [41], *Bacillus subtilis*, and *Salmonella enteritidis* [42], or *Staphylococcus aureus* [43], has been reported.

Most of the bacteria that represent the other genera identified in the present study were previously isolated from the digestive system of insects from the genus *Melolontha*. For example, *Serratia* sp., *Acinetobacter* sp., and *Citrobacter* sp. were isolated from the midgut of *M. hippocastani* L3 larvae [37], while a *Serratia marcescens* strain producing a highly active bacteriocin-like substance and *Acinetobacter* sp. have been isolated from *M. melolontha* [40,44]. In turn, *Chryseobacterium* sp. bacteria have been isolated from the gut of another member of the Scarabaeidae family, i.e., *Protaetia brevitarsis seulensis* [40]. Furthermore, the antibacterial activities of bacteria from the genera *Chryseobacterium* [45] and *Citrobacter* have been described before [46,47]; however, their interactions with the symbiotic bacteria of entomopathogenic nematode, have not been reported so far, as in the case of *Pseudomonas* or *Serratia*.

Importantly, 10 of the 12 selected isolates, i.e., representatives of the genera *Acinetobacter*, *Citrobacter*, *Pseudomonas*, and *Serratia*, belong to γ-proteobacteria, i.e., a class that comprises a number of human and animal pathogens, including many insect species. Various studies have reported the production of many compounds that inhibit the development of fungi, insects, and nematodes (e.g., phenazine-type antibiotics, hydrogen cyanide, chitinases, and proteases) by *P. chlororaphis* [48,49]. It has been evidenced that *P. chlororaphis* injected directly into the hemocoel caused the high mortality of *Galleria mellonella* (Lepidoptera: Pyralidae) larvae [50]. Oral and injectable toxicity to *Manduca sexta* (Lepidoptera: Sphingidae) and *Drosophila melanogaster* (Diptera: Drosophiladae) larvae have both also been described [51]. As shown by Schellenberger et al. [52], *P. chlororaphis* isolated from soil produces insecticidal protein, which is effective in *Diabrotica virgifera virgifera* (Coleoptera: Chrysomelidae) larvae. In turn, a *S. liquefaciens* strain that was isolated from *M. melolontha* larvae exerted a pathogenic effect on the larvae of *Dendroctonus micans* (Coleoptera: Curculionidae), *Thaumetopoea pityocampa* (Lepidoptera: Thaumetopoeidae), and *Lymantria dispar* (Lepidoptera: Erebidae) [53]. Similarly, *Chryseobacterium* sp. was shown to exhibit high pathogenicity to *D. melanogaster* when injected into the hemocoel [54].

Based on the literature data and the results of the present study, it can be concluded that the bacterial strains that are present in the *M. melolontha* larvae gut can potentially exert both adverse and beneficial effects on the health status of their hosts and on their survival under pressure from entomopathogenic nematodes. Consideration of the potential interactions between insect gut bacteria and symbiotic *Xenorhabdus* and *Photorhabdus* bacteria of entomopathogenic nematodes should address the question of whether these two groups of bacteria have a chance of mutual contact in natural conditions, i.e., in the insect organism. The natural environment for the development of *Xenorhabdus* spp. and *Photorhabdus* spp. is the hemocoel, where entomopathogenic nematodes, rather than the insect gut, release the bacteria. However, nematodes sometimes release their symbiotic bacteria still in the intestine, as mentioned earlier [12,16]. In such a case, it is possible that the bacteria carried by the nematode and those colonizing the insect can compete with each other in a direct manner. This suggests that such antagonism might be the insect’s first line of defense, being triggered before the immune response, which is initiated later when the bacteria enter the hemolymph. Importantly, a nematode must perforate the insect′s gut when penetrating from the alimentary canal to the hemocoel. While considering the size of bacterial cells, it might be assumed that the damaged gut becomes a gateway through which a certain amount of intestinal bacteria may enter the hemocoel. This seems to create good conditions for interactions between the two groups of bacteria at an early stage of infection. Furthermore, EPB, which find their way into the hemocoel proliferate at a fast rate, as this space provides optimal conditions for their growth, and the natural barriers that separate gut-associated bacteria from EPB, are quickly removed by bacterial enzymes that hydrolyze insect tissues, including the gut wall.

These preliminary investigations only provide a fragmentary description of the interactions between bacteria inhabiting the gut of scarab larvae and entomopathogenic nematode bacterial symbionts. Comprehensive research in this field was conducted with the use of different culture media, diverse screening methods for the determination of antibacterial activity, or a wider spectrum of insect and nematode species would certainly help to fully elucidate the mechanisms of bacterial interspecies competition and the insect defense against infection by entomopathogenic nematodes. Furthermore, a better understanding of the scarab larva-associated microbiota is necessary for elucidating the effect of natural microbiota on host resistance to pathogens. The identification of specific bacteria that protect insects from entomopathogenic nematodes might have great importance for manipulating insect fitness and susceptibility to pathogens, thus opening avenues for increasing the efficacy of pest management programs.

## 4. Materials and Methods

### 4.1. Entomopathogenic Nematodes

The investigations were carried out on five species of EPN that were isolated from the natural environment in Poland and identified with molecular methods. The nematodes were propagated in *Galleria mellonella* larvae. The infective larvae were collected after the nematodes have developed in the insect, while using the modified “white trap” method [55]. Straight genetic lines were derived from the isolates. Infective juveniles of EPN were kept in a sterile aqueous solution at 8 °C. The larvae were kept under these conditions for no longer than 15 days. The larvae of *G. mellonella* were grown on a natural diet. The experiments were conducted while using caterpillars weighing 180–200 mg.

### 4.2. Entomopathogenic Nematodes Symbiotic Bacteria

Five species of EPN bacterial symbionts were isolated from nematodes and used in the research (in the brackets names of the source nematodes): *X. bovienii* (*S. silvaticum*), *X. nematophila* (*S. carpocapsae*), *X. kozodoii* (*S. arenarium*), *X. budapestensis* (*S. bicornutum*), and *P. temperata* (*H. megidis*). The bacteria were stored frozen at −85 °C in lysogeny broth (LB) medium that was supplemented with 20% glycerol. Each time after thawing, the bacteria were tested on the NBTA, and blue or green colonies were used in subsequent analyses, since all of the experiments were exclusively based on EPB in the primary form.

### 4.3. Collection of M. melolontha Larvae from Their Natural Environment

*M. melolontha* L2 and L3 larvae were collected in forest and agricultural areas of the Lublin region (Eastern Poland). The collected grubs were put separately in plastic boxes with soil to prevent the insects from harmful biting. In the laboratory, the species and the grub stage of development were identified. Insects in good condition were selected for experiments and for the control group.

### 4.4. Exposure of M. melolontha to Selected EPN Species

The larvae of *M. melolontha* were placed separately in 150-mL plastic cups containing soil (−50 kPa water potential) from the larvae harvesting field. A scarab larva was placed at the bottom of each cup prior to addition of the soil. Infective juvenile stages of EPN were introduced in doses of 1000 IJs per insect. The cups were kept in incubators at 20 °C for 12 days.

### 4.5. Isolation of Bacteria from M. melolontha Midgut

All of the scarab larvae were surface sterilized with 70% alcohol, washed twice in sterile distilled water, and allowed to dry for 1 min. The digestive tract was dissected; then, the midgut was isolated and homogenized in 1 mL of 0.5% NaCl while using a glass tissue grinder. Serial 10-fold dilutions were spread on duplicated plates of LB agar and then incubated at 20 °C in aerobic or microaerobic (6% oxygen) conditions for 2–4 days. Single colonies were picked, purified by subculturing on plates, and then transferred to agar slants for further tests.

### 4.6. Antimicrobial Activity Assays

#### 4.6.1. Cross-Streak Tests

The nematode bacteria and the midgut isolates were grown separately in liquid LB medium for two days at 20 °C to prepare the inocula for the cross-streak tests. Each midgut isolate was then subinoculated as a middle line on a plate with LB agar medium. Each of all isolated *Xenorhabdus* or *Photorhabdus* strains was seeded in perpendicular lines on both sides of the midgut isolate line, and the plates were incubated at 20 °C for 72 h in aerobic or microaerophilic conditions. The antibacterial potential of gut isolates was measured (in mm) as an EFB inhibition zone.

#### 4.6.2. Modified Agar Well Diffusion Tests

The nematode bacteria and the midgut isolates were separately cultured in liquid LB medium for two days at 20 °C in the preparation of the inocula for the agar well diffusion assay. Next, warm (43 °C) LB medium containing 2% of agar placed in five conical flasks was separately inoculated with 2% of two-day old culture of nematode bacterial strains and then spilled (20 mL) onto disposable plastic Petri dishes with a 9-cm diameter. An appropriate number of wells (12 mm diameter) were cut out in the solidified agar medium. Each well was then filled with 260 μL of the warm LB medium with 2.0% agar inoculated with 2% of two-day old culture of a respective isolate strain. Two plates with one standard strain were intended for the study of its interaction with each midgut isolate. The plates were incubated at 20 °C for three days in the dark. The diameters (in mm) of the zones of inhibition of the nematode strain growth around the wells were determined.

#### 4.6.3. Maximum Inhibitory Dilution (MID) Tests

The isolates selected in previous tests were cultured in 100 mL Erlenmeyer flasks with 20 mL of LB medium. After 48 h of incubation at 20 °C, the cultures were centrifuged at 16,600× *g* for 15 min. The supernatants were filtered through sterile 0.22-μm nylon filters. Subsequently, series of two-fold dilutions of cell-free supernatants were prepared. At the same time, two-day-old cultures of EPB were suspended in sterile LB medium to a density of 0.5 McFarland standard. Afterwards, 100 μL of diluted supernatants and 100 μL of EPB suspensions were dispensed in a 96-well sterile microtiter plate and then incubated at 20 °C for 72 h. Afterwards, the growth of bacteria was measured spectrophotometrically at 600 nm (OD_600_) while using the Synergy HT Microplate Reader (Bio-Tek Instruments, Winooksi, VT, USA). All of the experiments were performed in two replicates, with five independent groups in each replication.

### 4.7. Molecular Identification of the Bacterial Isolates

Total genomic DNA was extracted while using a Genomic Mini AX Bacteria Spin Kit (A&A Biotechnology, Gdynia, Poland and then stored at −20 °C. All of the PCR amplifications were carried out with PCR mix RAPID (A&A Biotechnology, Gdynia, Poland), according to the manufacturer’s recommendations. The primer sequences and PCR conditions used are listed in Table 3. The amplified PCR products were purified with Clean-Up purification columns (A&A Biotechnology, Gdynia, Poland) and then sequenced in Genomed S.A. (Warsaw, Poland). Preliminarily, the 16S rRNA gene sequences were compared with the EzBioCloude database. All of the obtained sequences were analyzed while using BLAST available on the NCBI website. Multiple sequence alignment matrices of the individual gene sequences were created using ClustalW included in the MEGA 6.06 software [56]. The sequence identity values were calculated while using BioEdit 7.0.5 software.

The 16S rRNA gene sequence similarity threshold value of 98.7% between the isolate and species type strain was used as an indicator that an isolate can be a member of a given species [34]. The identification of the isolates at the species level was considered to be final when the searching results that were based on 16S rRNA gene sequences were concordant with those based on the *rpoB*, *rpoD*, or *recA* gene sequences.

### 4.8. Phylogenetic Analysis

Phylogenetic analyses of gene sequences were performed to confirm the identification results. The 16S rRNA, *rpoB*, *rpoD*, and *recA* gene sequences of isolates that were obtained in this study were compared to GenBank nucleotide sequences using BLAST (NCBI). Multiple sequence alignments were created using ClustalW at the default configuration and manually checked. The evolutionary distances were computed using the Tamura–Nei algorithm and the phylogenetic trees were generated using the neighbor-joining method in MEGA 6.06. All of the positions containing gaps and missing data were eliminated. Bootstrapping with 1000 replicates of the data was conducted to determine the statistical support for the branches.

All of the gene sequences that were obtained in this study were deposited in the GeneBank database under the accession numbers given in Table 4 and depicted in the phylogenetic trees (Supplementary Materials Figures S1–S6).

### 4.9. Statistical Analysis

The data were pooled before statistical analysis since there were no statistically significant differences between the results of the replicates of all experiments. The test results were subjected to one-way ANOVA and Tukey′s post-hoc tests (supernatant dilutions). The t-student test was used for pairwise comparisons between bacteria species. The normality of the data distribution was determined while using the Shapiro–Wilk test and the homogeneity of variance was assessed by the Levene test. The occurrence of statistically significant differences in these experiments was based on the overlap of 95% confidence intervals. Differences among means were considered to be significant at *p* < 0.05. All of the statistical analyses were performed while using the IBM SPSS Statistics 24 software package.

## Figures and Tables

**Table 1:** Antagonistic activities of selected *M. melolontha* midgut isolates against five entomopathogenic bacteria (EPB) strains shown by cross-streak tests.

Strain ID *	Size of Inhibition Zones (mm) Left Inhibition Zone|Right Inhibition Zone				
	*P. temperata*	*X. kozodoii*	*X. bovienii*	*X. nematophila*	*X. budapestensis*
MT1 L2 01	0|0	12|13	20|18	no gr. **| no gr.	no gr.|no gr.
MT1 L2 02	1|1	1|1	no gr.|no gr.	no gr.|no gr.	7|4
MT3 L2 02	2|4	no gr.|no gr.	no gr.|no gr.	no gr.|no gr.	no gr.|no gr.
MT3 L2 05	12|7	no gr.|no gr.	no gr.|no gr.	no gr.|no gr.	no gr.|no gr.
MT3 L2 06	6|6	no gr.|no gr.	no gr.|no gr.	no gr.|no gr.	no gr.|no gr.
MT3 L2 08	10|12	no gr.|no gr.	no gr.|no gr.	no gr.|no gr.	no gr.|no gr.
MT3 L2 10	12|11	no gr.|no gr.	no gr.|no gr.	no gr.|no gr.	no gr.|no gr.
MT3 L2 13	7|5	no gr.|no gr.	no gr.|no gr.	no gr.|no gr.	no gr.|no gr.
MT4 L2 11	0|0	0|0	30|22	no gr.|24	18|no gr.
MTA1 L2 01	1|1	no gr.|no gr.	no gr.|12	6|6	30|20
MTA3 L2 13	5|5	25|25	no gr.|no gr.	no gr.|no gr.	no gr.|no gr.
MTA5 L3 10	3|5	7|5	no gr.|no gr.	no gr.|no gr.	20|25
MMB2 L3 03	0|0	0|1	11|12	4|0	3|3
MMB2 L3 04	0|0	0|0	7|6	0|0	1| 0
MMB2 L3 07	0|0	0|0	12|12	9|10	10|9
MMB4 L3 10	0|0	0|0	11|12	1|0	0|0
MTB1 L3 08	0|0	15|14	no gr.|no gr.	no gr.|no gr.	no gr.|no gr.
MTB1 L3 09	0|0	15|20	no gr.|no gr.	no gr.|no gr.	no gr.|no gr.
MTB1 L3 12	0|0	no gr.|no gr.	no gr.|no gr.	no gr.|no gr.	no gr.|no gr.
MTB5 L3 11	0|0	7|10	no gr.|no gr.	no gr.|no gr.	17|22
MMC3 L3 03	0|0	11|12	14|16	14|13	12|13
MMC3 L3 04	0|0	11|13	15|17	15|17	15|14
MMC3 L3 07	0|0	13|14	16|18	17|19	17|17
MMC3 L3 12	0|0	12|14	15|17	15|16	15|14
MTC1 L3 03	0|0	10|10	no gr.|no gr.	no gr.|no gr.	no gr.|no gr.
MTS3 L3 15	8|7	25|25	no gr.|no gr.	no gr.|no gr.	no gr.|no gr.
MMH3 L2 04	2|2	3|2	17|18	17|16	13|14
MMH3 L2 05	2|1	3|2	14|16	12|15	12|10
MMH3 L2 06	2|1	4|3	16|17	16|15	11|13
MMH4 L2 06	1|1	2|3	17|15	10|13	9 |12
MMH4 L2 07	1|1	2|2	14|13	12|10	12|11
MMH5 L2 04	0|1	2|3	2|3	1|1	7|5
MMH5 L2 08	0|0	0|0	4|4	5|5	3|3
MTH3 L2 08	0|0	16|14	no gr.|no gr.	no gr.|no gr.	no gr.|no gr.
MTH3 L2 09	0|0	10|9	no gr.|no gr.	no gr.|no gr.	no gr.|no gr.
MTH3 L2 10	0|0	10|10	no gr.|no gr.	no gr.|no gr.	no gr.|no gr.
MTH3 L2 14	0|0	14|15	no gr.|no gr.	no gr.|no gr.	no gr.|no gr.
MTH3 L2 15	0|0	10|10	no gr.|no gr.	no gr.|no gr.	no gr.|no gr.

* Strain ID provides information about the conditions of the isolation and the most important data on the *M. melolontha* larva used for isolation thereof. The second letter in strain ID: M—isolation in microaerobic conditions; T—isolation in aerobic conditions. The third letter in strain ID: information about the initial exposure of the *M. melolontha* larva to: A—*S. arenarium*; B—*S. bicornutum*; C—*S. carpocapsae*; S—*S. silvaticum*; H—*H. megidis*. L2 or L3: developmental stage of the *M. melolontha* larva. ** no gr.—no growth. Complete inhibition of bacterial strain growth.

**Table 2:** Antagonistic activities of selected *M. melolontha* midgut isolates against five EPB strains that were shown by modified agar well diffusion tests.

Strain ID	Diameter of the Inhibition Zone (mm)				
	*P. temperata*	*X. kozodoii*	*X. bovienii*	*X. nematophila*	*X. budapestensis*
**MT3 L2 05**	0.00	0.00	14.00 ± 0.41	15.85 ± 0.41	15.35 ± 0.58
**MT3 L2 08**	14.10 ± 0.46	16.95 ± 0.64	0.00	17.05 ± 0.55	17.45 ± 0.55
**MT3 L2 10**	15.00 ± 0.33	18.55 ± 0.64	0.00	18.05 ± 0.64	17.05 ± 0.50
**MT3 L2 13**	0.00	0.00	14.00 ± 0.58	16.05 ± 0.28	14.95 ± 0.44
**MTA1 L2 01**	13.9 ± 0.66	0.00	15.05 ± 0.59	14.45 ± 0.39	0.00
**MTA3 L2 13**	0.00	0.00	0.00	0.00	19.20 ± 0.48
**MTB1 L3 12**	0.00	17.10 ± 0.57	0.00	0.00	18.05 ± 0.28
**MTC1 L3 03**	0.00	0.00	0.00	0.00	19.25 ± 0.63
**MMC3 L3 04**	16.00 ± 0.41	18.95 ± 0.60	16.10 ± 0.39	16.15 ± 0.34	19.90 ± 0.70
**MMH3 L2 04**	0.00	16.05 ± 0.44	15.40 ± 0.32	17.05 ± 0.37	17.45 ± 0.39
**MMH4 L2 06**	0.00	15.50 ± 0.52	0.00	17.00 ± 0.58	16.45 ± 0.55
**MTS3 L3 15**	0.00	17.95 ± 0.68	14.05 ± 0.37	18.45 ± 0.44	16.10 ± 0.57

All data are expressed as mean ± SD, *n* = 10.

**Table 3:** Oligonucleotides used in the study.

Primer	Sequence	Target Gene	Target Bacterial Genus	PCR Cycling	Product Length	Reference
27F 1492R	5′-AGAGTTTGATCCTGGCTCAG-3′ 5′-GGTTACCTTGTTACGACTT-3′	16S rDNA	All tested	3 min 95 °C, 30 × (30 s 94 °C, 45 s 55 °C, 90 s 72 °C), 5 min 72 °C	1500 bp	[21]
PsEG30F PsEG790R	5′- ATYGAA ATCGCCAARCG-3′ 5′-CGGTTGATKT CCTTGA-3′	*rpoD*	*Pseudomonas*	3 min 95 °C, 30 × (60 s 94 °C, 45 s 55 °C, 50 s 72 °C), 5 min 72 °C	750 bp	[22]
Vic3 Vic2	5′-GGCGAAATGGCWGAGAACCA-3′ 5′-GAGTCTTCGAAGTTGTAACC-3′	*rpoB*	*Citrobacter*	4 min 94 °C, 30 × (30 s 94 °C, 30 s 50 °C, 45 s 72 °C), 5 min 72 °C	410 bp	[23]
Ac696F Ac1093R	5′-TAYCGYAAAGAYTTGAAAGAAG-3′ 5′-CMACACCYTTGTTMCCRTGA-3′	*rpoB*	*Acinetobacter*	3 min 95 °C, 30 × (60 s 94 °C, 52 s 45 °C, 60 s 72 °C), 5 min 72 °C	370 bp	[24]
359f 359r	5′-TTATCGCTCAGGCGAACTCCAAC-3′ 5′-TGCTGGATTCGCCTTTGCTACG-3′	*rpoB*	*Serratia*	3 min 95 °C, 30 × (50 s 94 °C, 40 s 52 °C, 60s 72 °C), 5 min 72 °C	530 bp	[25]
ESchr-rpoF ESchr-rpoR	5′GGTGAAGTAGTTTCTATCGAAAGA-3′ 5′-ATGTTTGGTCCTTCCGGAGTT-3′	*rpoB*	*Chryseobacterium*	3 min 95 °C, 30 × (35 s 95 °C, 35 s 52 °C, 50 s 72 °C), 5 min 72 °C	790 bp	This work
recAF recAR	5′-TCSGGYAARACCACSCTGAC-3′ 5′-RTACCAGGCRCCGGACTTCT-3′	*recA*	*Pseudomonas*	4 min 94 °C, 30 × (30 s 94 °C, 30 s 55 °C, 40 s 72 °C), 5 min 72 °C	600 bp	[26]

**Table 4:** Molecular identification of bacterial strains with antimicrobial activity against EPB isolated from the midgut of *M. melolontha* larvae.

Strain ID	Identification Result/Gene Accession Numbers	Strain with the Highest Similarity to the Isolate in the Gene Bank Based on the Gene Sequence/Gene Accession Number/% Nucleotide Identity		
		16S rDNA	*rpoD/rpoB*	*recA*
**MT3** **L2 05**	*Pseudomonas chlororaphis* 16S rDNA—MM421924 *rpoD*—MN445046	*P. chlororaphis* subsp. *piscum* JF3835^T^ (FJ168539)—99.8%	* *P. chlororaphis* subsp. *piscum* ZJU60 (CP027656)—98.2%; *P. chlororaphis* subsp. *aurefaciens* NCIMB 9265 (AB039555)—98.2%	n.d.
**MT3** **L2 08**	*Pseudomonas chlororaphis* 16S rDNA—MM421925 *rpoD*—MN445047	*P. chlororaphis* subsp. *piscum* JF3835^T^ (FJ168539)—99.8%	* *P. chlororaphis* subsp. *piscum* ZJU60 (CP027656)—98.2%; *P. chlororaphis* subsp. *aurefaciens* NCIMB 9265 (AB039555)—98.2%	n.d.
**MT3** **L2 10**	*Pseudomonas chlororaphis* 16S rDNA—MM421926 *rpoD*—MN445048 *recA*—MN477250	*P. chlororaphis* subsp. *piscum* JF3835^T^ (FJ168539)—99.8%	* *P. chlororaphis* subsp. *piscum* ZJU60 (CP027656)**—98.0%; *P. chlororaphis* subsp. *aurefaciens* NCIMB 9265 (AB039555)—98.0%	*P. chlororaphis* subsp*. piscum* JF3835^T^ (FJ168540)**—98.4%
**MT3** **L2 13**	*Pseudomonas chlororaphis* 16S rDNA—MM421923 *rpoD*—MN445049	*P. chlororaphis* subsp. *piscum* JF3835^T^ (FJ168539)—99.9%	* *P. chlororaphis* subsp. *piscum* ZJU60 (CP027656)—98.0%; *P. chlororaphis* subsp. *aurefaciens* NCIMB 9265 (AB039555)—98.0%	n.d.
**MTA1** **L2 01**	*Citrobacter murlinae* 16S rDNA—MM421947 *rpoB*—MN445055	*C. murliniae* CIP 104556^T^ (KY178281)—99.3%	** *C. murliniae* CIP 104556^T^ (KM516007)—99.5%	n.d.
**MMC3** **L3 04**	*Pseudomonas chlororaphis* 16S rDNA—MM421927 *rpoD*—MN445050 *recA*—MN477251	*P. chlororaphis* subsp. *piscum* JF3835^T^ (FJ168539)—99.8%	* *P. chlororaphis* subsp. *piscum* ZJU60 (CP027656)—98.2%; *P. chlororaphis* subsp. *aurefaciens* NCIMB 9265 (AB039555)—98.2%	*P. chlororaphis* subsp*. piscum* JF3835^T^ (FJ168540)**—98.6%
**MTB1** **L3 12**	*Serratia liquefaciens* 16S rDNA—MM422010 *rpoB*—MN445052	*Serratia quinivorans* DSM4597^T^ (AJ233435)—99.4%; *S. liquefaciens* CIP 103238^T^ (NR 042062)—98.9%	** *S. liquefaciens* LMG7884^T^ (JX425335)—99.4% *S. quinivorans* LMG7887^T^ (JX425309)—97.7%	n.d.
**MTA3** **L2 13**	*Acinetobacter calcoaceticus* 16S rDNA—MN429316 *rpoB*—MN445056	*A. calcoaceticus* DSM 30006^T^ (AJ633632)—99.8%	** *A. calcoaceticus* CIP 81.8^T^ (DQ207474)—97.9%; *A. calcoaceticus* CA16 (NZ_CP020000)—98.7%	n.d.
**MTS3 L3 15**	*Pseudomonas chlororaphis* 16S rDNA—MM421928 *rpoD*—MN445051 *recA*—MN477252	*P. chlororaphis* subsp. *piscum* JF3835^T^ (FJ168539)—99.7%	* *P. chlororaphis* subsp. *piscum* ZJU60 (CP027656)—98.4%; *P. chlororaphis* subsp. *aurefaciens* NCIMB 9265—98.4%	*P. chlororaphis* subsp*. piscum* JF3835^T^ (FJ168540)**—98.6%
**MTC1** **L3 03**	*Serratia* sp. 16S rDNA—MM422009 *rpoB*—MN445053	*Serratia grimesii* DSM 30063^T^ (AJ233430)—99.3%; *Serratia proteamaculans* DSM 4543^T^ (AJ233434)—99.2% *S. liquefaciens* CIP 103238 (AJ306725)—99.1%	** *S. liquefaciens* LMG7884^T^ (JX425335)—98.8% *S. quinivorans* LMG7887^T^ (JX425309)—98.3%	n.d.
**MMH3** **L2 04**	*Chryseobacterium lathyri* 16S rDNA—MM429317 *rpoB*—MN445063	*C. lathyri* RBA2-6^T^ (DQ673674)—99.8%	** *C. lathyri* KCTCT 22544^T^ (NZ_QNFY01000004)—99.1%	n.d.
**MMH4** **L2 06**	*Chryseobacterium* sp. 16S rDNA—MN429318 *rpoB*—MN445064	*C. nakagawai* NCTC 13529^T^ (NZLR134386)—98.7%	** *C. joostei* DSM 16927^T^ (CP033926)—93.6%; *C. nakagawai* NCTC 13529^TT^ (LR134386)—91.0%	n.d.

n.d.—not determined, * *rpoD* gene sequences analysis; ** *rpoB* gene sequences analysis.

## Supplementary Materials

The following are available online at https://www.mdpi.com/1422-0067/21/2/580/s1, Figure S1: Neighbor joining tree based on 16S rRNA gene sequences showing the phylogenetic relationships of the studied *M. melolontha* midgut isolates (bolded) exhibiting antagonistic activity against bacterial symbionts of entomopathogenic nematodes., Figure S2 Neighbor joining tree based on *rpoD* gene sequences showing the phylogenetic relationships of the studied *M. melolontha* midgut *Pseudomonas* isolates (bolded) exhibiting antagonistic activity against bacterial symbionts of entomopathogenic nematodes., Figure S3: Neighbor joining tree based on *rpoB* gene sequences showing the phylogenetic relationships of the studied *M. melolontha* midgut *Serratia* and *Citrobacter* isolates (bolded) exhibiting antagonistic activity against bacterial symbionts of entomopathogenic nematodes., Figure S4: Neighbor joining tree based on *rpoB* gene sequences showing the phylogenetic relationships of the studied *M. melolontha* midgut *Acinetobacer* isolate (bolded) exhibiting antagonistic activity against bacterial symbionts of entomopathogenic nematodes., Figure S5: Neighbor joining tree based on *rpoB* gene sequences showing the phylogenetic relationships of the studied *M. melolontha* midgut *Chryseobacterium* isolates (bolded) exhibiting antagonistic activity against bacterial symbionts of entomopathogenic nematodes., Figure S6: Neighbor joining tree based on *recA* gene sequences showing the phylogenetic relationships of the studied *M. melolontha* midgut *Pseudomonas* isolates (bolded) exhibiting antagonistic activity against bacterial symbionts of entomopathogenic nematodes.

## Abbreviations

ANOVA	Analysis of variance
BLAST	Basic local alignment search tool
EPB	Entomopathogenic bacteria
EPN	Entomopathogenic nematodes
IJs	Infective juveniles
LB	Lysogeny broth (or Luria broth)
MID	Maximum inhibitory dilution
NBTA	Nutrient bromothymol blue agar
NCBI	National Center for Biotechnology Information
PCR	Polymerase chain reaction
