# Supplementary material for: Bacteria from the Midgut of Common Cockchafer (Melolontha melolontha L.) Larvae Exhibiting Antagonistic Activity Against Bacterial Symbionts of Entomopathogenic Nematodes: Isolation and Molecular Identification
Source: Int J Mol Sci. 2020 Jan 16;21(2):580. doi: 10.3390/ijms21020580 (PMC7013910; doi:10.3390/ijms21020580)
Supplement: Supplementary file 1 [file ijms-21-00580-s001.pdf]

Supplementary Data

Article

Bacteria from the Midgut of Common Cockchafer (*Melolontha melolontha* L.) Larvae Exhibiting Antagonistic Activity Against Bacterial Symbionts of Entomopathogenic Nematodes: Isolation and Molecular Identification

Marcin Skowronek [1,*], Ewa Sajnaga [1], Małgorzata Pleszczyńska [2], Waldemar Kazimierczak [1], Magdalena Lis [1] and Adrian Wiater [2,*]

[1] Laboratory of Biocontrol, Application and Production of EPN, Centre for Interdisciplinary Research, Faculty of Biotechnology and Environmental Sciences, John Paul II Catholic University of Lublin, ul. Konstantynów 1J, 20-708 Lublin, Poland; esajnaga@kul.pl (E.S.); wklublin@tlen.pl (W.K.); mdybala@kul.pl (M.L.)

[2] Department of Industrial and Environmental Microbiology, Faculty of Biology and Biotechnology, Maria Curie-Skłodowska University, ul. Akademicka 19, 20-033 Lublin, Poland; mpleszcz@poczta.umcs.lublin.pl

* Correspondence: marskow@kul.pl (M.S.); adrianw2@poczta.umcs.lublin.pl (A.W.)

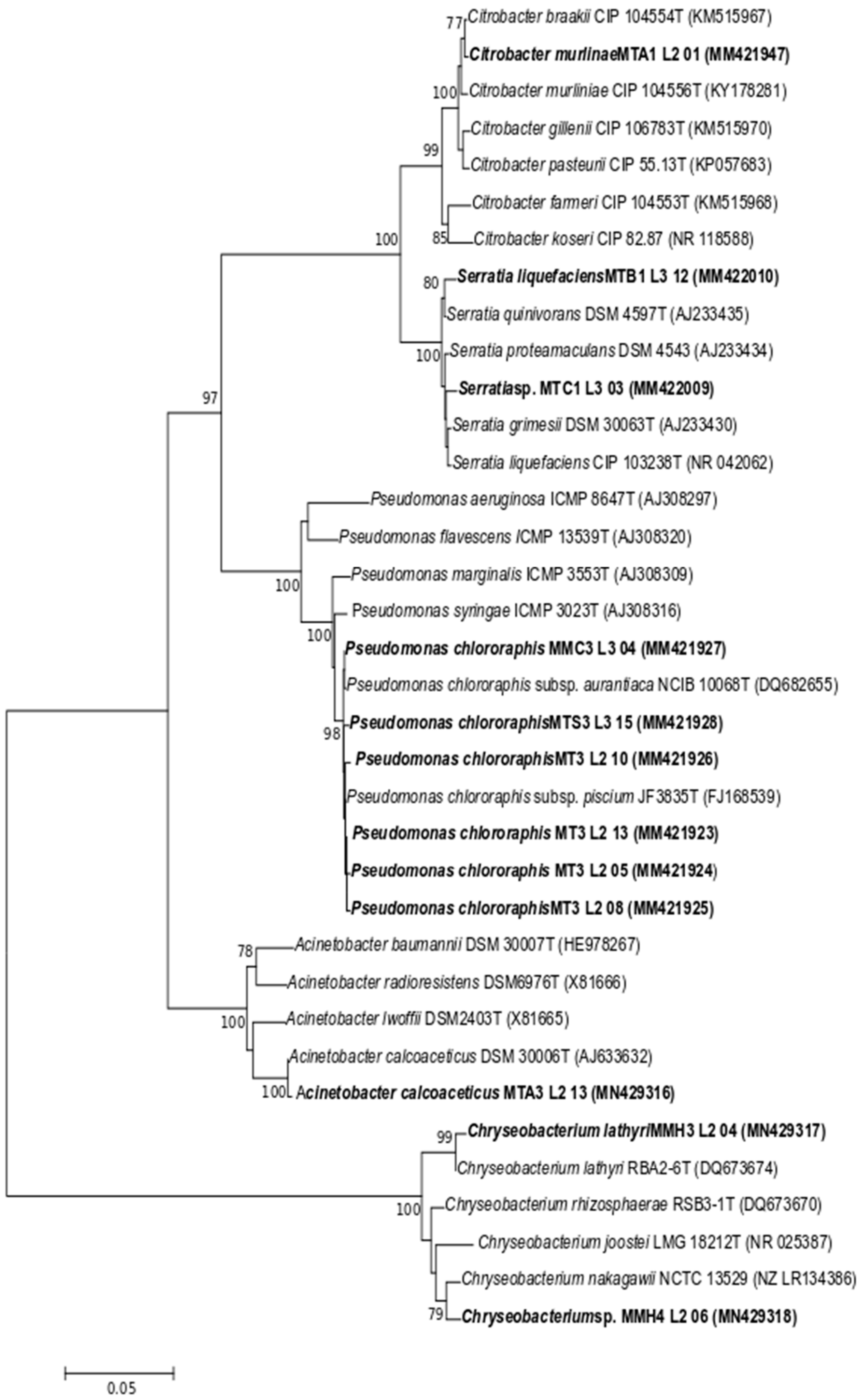

Figure S1. Neighbor joining tree based on 16S rRNA gene sequences showing the phylogenetic relationships of the studied *M. melolontha* midgut isolates (bolded) exhibiting antagonistic activity against bacterial symbionts of entomopathogenic nematodes. The GeneBank accession numbers are given in parentheses. The numbers at the branching points are bootstrap values >50%. The bar indicates 0.05 substitution per nucleotide positions.

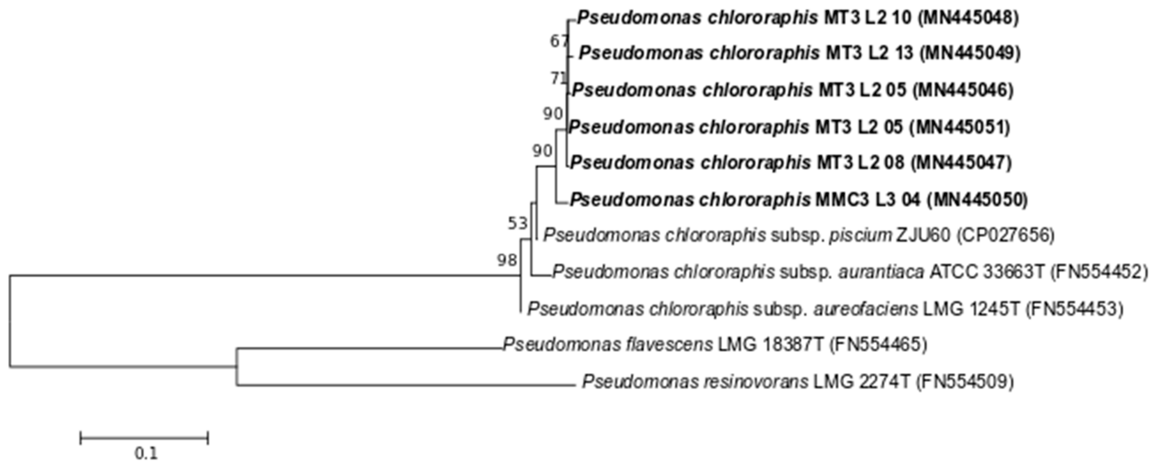

Figure S2. Neighbor joining tree based on *rpoD* gene sequences showing the phylogenetic relationships of the studied *M. melolontha* midgut *Pseudomonas* isolates (bolded) exhibiting antagonistic activity against bacterial symbionts of entomopathogenic nematodes. The GeneBank accession numbers are given in parentheses. The numbers at the branching points are bootstrap values >50%. The bar indicates 0.01 substitution per nucleotide positions.

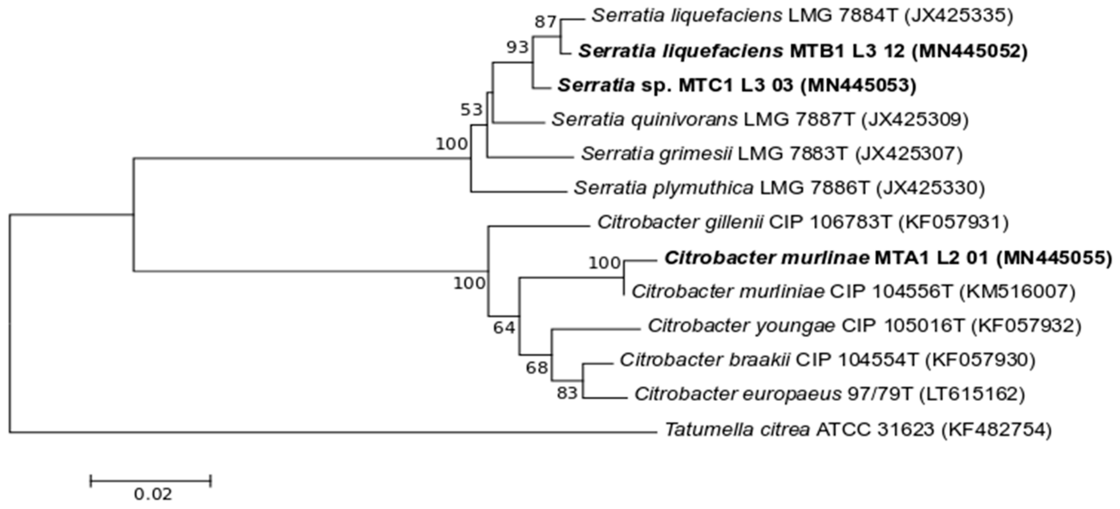

Figure S3. Neighbor joining tree based on *rpoB* gene sequences showing the phylogenetic relationships of the studied *M. melolontha* midgut *Serratia* and *Citrobacter* isolates (bolded) exhibiting antagonistic activity against bacterial symbionts of entomopathogenic nematodes. The GeneBank accession numbers are given in parentheses. The numbers at the branching points are bootstrap values >50%. The bar indicates 0.02 substitution per nucleotide positions. The sequence of *Tatumella citrea* was used as an outgroup.

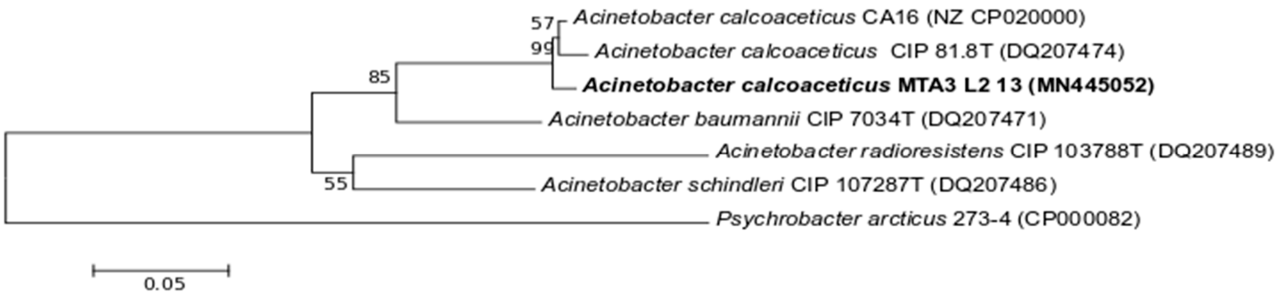

Figure S4. Neighbor joining tree based on *rpoB* gene sequences showing the phylogenetic relationships of the studied *M. melolontha* midgut *Acinetobacer* isolate (bolded) exhibiting antagonistic activity against bacterial symbionts of entomopathogenic nematodes. The GeneBank accession numbers are given in parentheses. The numbers at the branching points are bootstrap values >50%. The bar indicates 0.05 substitution per nucleotide positions. The sequence of *Psychrobacter arcticus* was used as an outgroup.

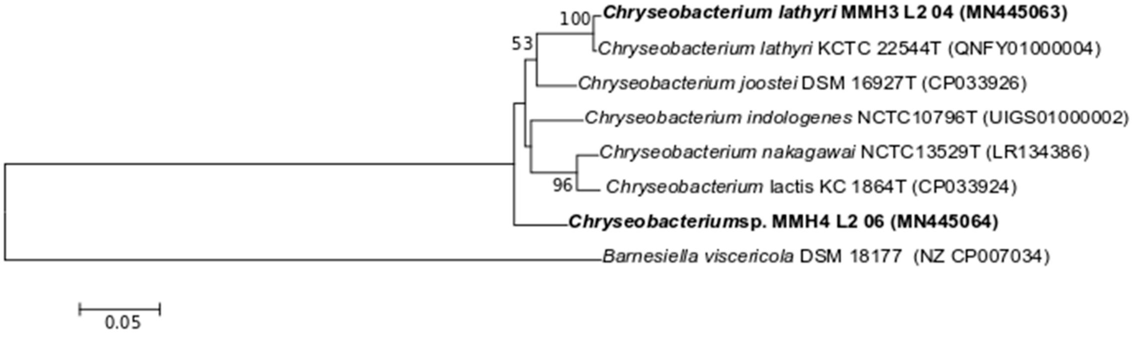

Figure S5. Neighbor joining tree based on *rpoB* gene sequences showing the phylogenetic relationships of the studied *M. melolontha* midgut *Chryseobacterium* isolates (bolded) exhibiting antagonistic activity against bacterial symbionts of entomopathogenic nematodes. The GeneBank accession numbers are given in parentheses. The numbers at the branching points are bootstrap values >50%. The bar indicates 0.05 substitution per nucleotide positions. The sequence of *Bamesiella viscericola* was used as an outgroup.

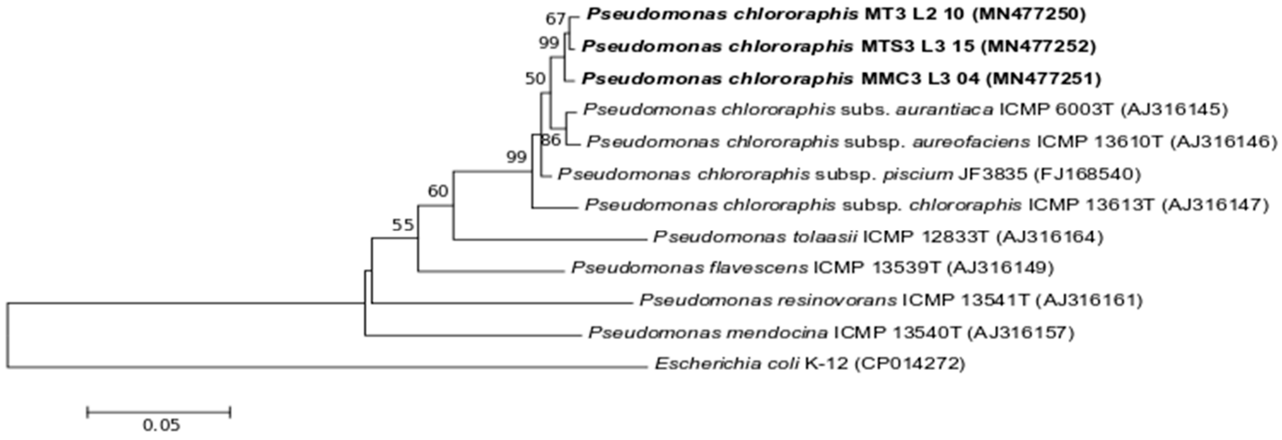

Figure S6. Neighbor joining tree based on *recA* gene sequences showing the phylogenetic relationships of the studied *M. melolontha* midgut *Pseudomonas* isolates (bolded) exhibiting antagonistic activity against bacterial symbionts of entomopathogenic nematodes. The GeneBank accession numbers are given in parentheses. The numbers at the branching points are bootstrap values >50%. The bar indicates 0.05 substitution per nucleotide positions. The sequence of *Escherichia coli* was used as an outgroup.
